# Decoding the mysteries of aging and its impact on human health

**DOI:** 10.52601/bpr.2023.230902

**Published:** 2023-10-31

As we age, we also move towards aging. But the seemingly harmonious and peaceful organism is actually far harder than we think. Aging is the root cause of many diseases. This special issue gathers experts from the Chinese Academy of Sciences, Peking University, Tsinghua University, South China Normal University, and Hangzhou Normal University, *etc*.; for example, Li Peng, Liu Guanghui, Yu Li, Gao Chengjiang, Cong Yusheng, *etc*., the latest research review and experimental methods in this field are aimed at in-depth exploration of the internal relationship between immunity, aging, regenerative medicine and others, with a view to providing strong support for the development of human health.

This special issue contains the research results of many well-known domestic experts and scholars, covering the fields of immunity, stem cells, aging and aging-related diseases, involving gene signal transduction, artificial intelligence, gene editing, stem cell regenerative medicine and other aspects. Such as innate immune pathways, such as cGAS-STING, NF-κB, and NLRP3 inflammasome, participating in the senescence process. They also propose strategies by which we can improve the immune function and reduce inflammation using the above findings. These results not only reveal the inherent mechanism of aging and disease, but also provide new ideas and methods for anti-aging, anti-inflammatory, prevention and therapy of diseases.

Although some achievements have been made in aging, regenerative medicine and disease research, there are still many unknown areas that need to be further explored. This special issue also looks forward to future research directions, including interdisciplinary collaboration, application of new technologies, and clinical trials. We believe that with the deepening of research, human beings will better understand the mysteries of aging, regenerative medicine and various diseases of the body, and make greater contributions to the health and well-being of all mankind. For researchers, this special issue provides important scientific information in the field of aging and disease research, and for ordinary researchers who want to understand aging and how to study aging and regeneration research, this special issue is also worth reading.

In order to provide our readers with a more comprehensive understanding of this complex and multifaceted topic, we have decided to publish this special issue in three parts. Each part will delve into different aspects of aging and its impact on human health, offering our readership an extensive array of insights and research outcomes.

## Conflict of interest

 declare that they have no conflict of interest.

